# Nitrile Groups as Build-In Molecular Sensors for Interfacial Effects at Electrocatalytically Active Carbon–Nitrogen Materials

**DOI:** 10.1021/acsami.5c02366

**Published:** 2025-04-09

**Authors:** Linda Feuerstein, Ekin Esme Bas, Dorothea Golze, Thomas Heine, Martin Oschatz, Inez M. Weidinger

**Affiliations:** † Chair of Electrochemistry, Technische Universität Dresden, Zellescher Weg 19, Dresden 01069, Germany; ‡ Chair of Theoretical Chemistry, Technische Universität Dresden, Bergstrasse 66c, Dresden 01069, Germany; § Helmholtz-Zentrum Dresden-Rossendorf, HZDR, Bautzner Landstrasse 400, Dresden 01328, Germany; ∥ Center for Advanced Systems Understanding, CASUS, Untermarkt 20, Görlitz 02826, Germany; ⊥ Department of Chemistry, Yonsei University and ibs-cnm, Seodaemun-gu Seoul 120-749, Republic of Korea; # Center for Energy and Environmental Chemistry, Friedrich Schiller University Jena, Philosophenweg 7a, Jena 07743, Germany; ∇ Institute for Technical Chemistry and Environmental Chemistry, Friedrich Schiller University Jena, Philosophenweg 7a, Jena 07743, Germany; ○ Helmholtz Institute for Polymers in Energy Applications Jena (HIPOLE Jena), Lessingstraße 12−14, Jena 07743, Germany

**Keywords:** Raman spectroscopy, electrochemical double layer, carbon−nitrogen material, Vibrational Stark Effect, hydrogen evolution, local electric fields

## Abstract

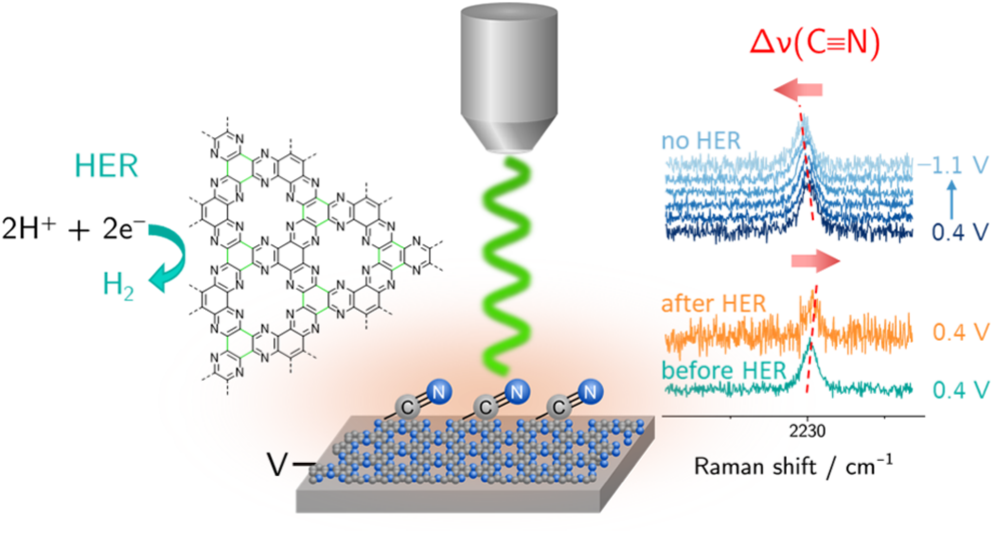

Electrocatalytic reactions are influenced by various interfacial phenomena including nonspecific interaction forces. For many examples, their contributions to the catalytic cycle have yet to be identified. Noncovalent interactions between the electrode and the electrolyte can be described by the local electric field environment at the interface and are experimentally accessible based on the Vibrational Stark Effect. We herein present a carbon-based C_2_N-type electrocatalyst that is active for the hydrogen evolution reaction and that contains nitrile functions as Stark reporter groups. With this system, we expand the range of electrocatalytically active systems suitable for electrochemical Stark spectroscopy while taking a step away from pure model systems. The stretching mode ν(C≡N) was analyzed via experimental and calculated Raman spectroscopy, revealing a defect character of the inherent CN groups. The ν(C≡N) peak position was furthermore studied via in situ electrochemical Raman spectroscopy. At noncatalytic conditions, a linear dependence between an applied electric potential and ν(C≡N) peak shift is observed, resulting in a red-shift at a more negative potential. At catalytic conditions, deviations from the linearity occur, and a semipermanent blue-shift of the CN peak is observed after electrocatalysis, implying a restructuring of the electrochemical double layer and therefore a change in the local electric field environment due to the catalytic turnover and the associated interfacial processes.

## Introduction

The course of a chemical reaction under ambient conditions is mainly controlled by molecular interactions of the involved chemical species. Next to covalent bonding, weaker forces such as H-bonds, dipole–dipole, and van der Waals interactions mainly determine the kinetically favored route and the reaction rate. In order to quantify and compare these noncovalent forces as well as to estimate interaction and stabilization energies, a universal unit needs to be defined. Instead of a full quantum-mechanical treatment of the whole system, the description of the chemical environment by means of an electric (vector) field has proven to be a suitable and versatile approach for unifying the different types of interaction forces into one quantity, as suggested by Fried and Boxer.^1,2^ More specifically, the transposition of a quantum-mechanical description into an electrostatic one is performed, where the molecule under consideration is represented by a dipole (or collection of dipoles)  within an electric field , created by all charges and dipoles of the chemical environment, resulting in the interaction energy .^1,2^ For example, this concept was successfully applied to demonstrate the working principles of enzymatically catalyzed reactions. It was shown that large electric field environments of up to 1.4 V/Å are present within the active sites of enzymes, created by the functional groups of the encoded amino acids.^3,4^ In addition, the favorable effect of polar solvents on S_N_1-type organic reactions,^5,6^ as well as the influence of an oriented external electric field that is applied in field-assisted electrostatic catalysis, can be accurately explained using the electric field language.^7−10^

Electrochemical catalysis is the basis for many modern energy conversion technologies, and an external electric field is omnipresent here in the form of a polarizable electrode that creates an electrostatic field across the electrode–electrolyte interface. This field is screened by the charges present in the electrochemical double layer (ECDL), mainly the Helmholtz layer, which reaches thicknesses on the order of 10^0^–10^1^ Å. At voltages around 1 V, which are common in practice, the magnitude of the resulting electric field can therefore be roughly estimated at 0.1 to 1 V/Å in most electrochemical systems.^11,12^ Upon closer examination, the effective electric field environment for each individual system is strongly determined by the exact composition of the ECDL. The dipoles (their magnitude and orientation) modulate the incident electric field from the electrode and generate local electric fields. Many mechanistic investigations on electrocatalytic charge-transfer reactions highlight the importance of the formation of energetically stabilized intermediates for catalytic efficiency.^13−15^ In contrast, the mechanistic influence of these local electric fields and the contributions originating from the electrostatic effects of the charged electrode, the solvation field of the ECDL, and noncovalent interactions of the present species within the interfacial region is not fully understood yet and much less frequently discussed.^16−20^ Monitoring and characterizing these conditions and clarifying both beneficial and hindering effects on the performance of electrocatalytic systems are a key focal point in current energy conversion research.^21−26^

A major problem in analyzing electric field effects on reaction dynamics is that local electric fields are difficult to measure and can also change during the reaction. One method to study local electric fields is based on the vibrational Stark effect (VSE).^1,27^ The VSE describes the interaction between the local electric field and the vibrational energy levels of a molecule within that field, resulting in an experimentally observed vibrational frequency shift Δν. In the conventional physical description of the linear VSE, Δν is expressed by the scalar product of the local electric field vector  and the difference dipole moment  of the respective vibration:^1,27^
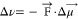
1

On this basis, in situ vibrational spectroscopy on heterogeneous systems containing Stark reporter groups is a versatile tool for investigating electric field effects at the solid–liquid interface.^17,26,28−33^ By what is sometimes called “electrochemical Stark spectroscopy,” the vibrational frequency shifts of surface-bound species are measured as a function of applied potential via in situ spectroscopical techniques such as Raman, infrared (IR), or vibrational sum frequency generation (SFG) spectroscopy.^17,28−35^ Here, the −C≡N triple bond functionality has proven to be a strong Stark reporter group because the characteristic group frequency of the stretching vibration ν(C≡N) falls within a spectral range where few other signals are found, typically at around 2220–2250 cm^–1^. Recent discussions have been opened regarding an alternative interpretation of the observed vibrational frequency shifts, which highlight the influence of “through-bond effects” on the peak positions of Stark reporter groups as opposed to “through-space effects” based solely on electrostatic fields.^31−33^ Arguably, applying potential to an electrode could lead to inductive effects caused by the electrode through electron-donating (+I) or -withdrawing effects (−I) similar to the picture drawn for functional groups in classical organic chemistry.^32^ Accordingly, these electroinductive effects could provide a different explanation for peak shifts in the context of electrochemical systems.

To date, most studies on electrode–electrolyte interfaces are focused on model systems, often featuring noble metal surfaces functionalized with self-assembled monolayers (SAMs) containing nitrile groups.^17,26,28−32,35,36^ Furthermore, interest has expanded to study energy conversion reactions with electrochemical Stark spectroscopy including electrocatalytic reactions such as hydrogen evolution reaction (HER) and carbon dioxide reduction reaction (CO_2_RR).^29,34,37^ However, studies in this area have also concentrated on model systems, which are far from practical implementation in future energy conversion technologies due to the high costs associated with noble-metal-based systems.

**Figure 1:**
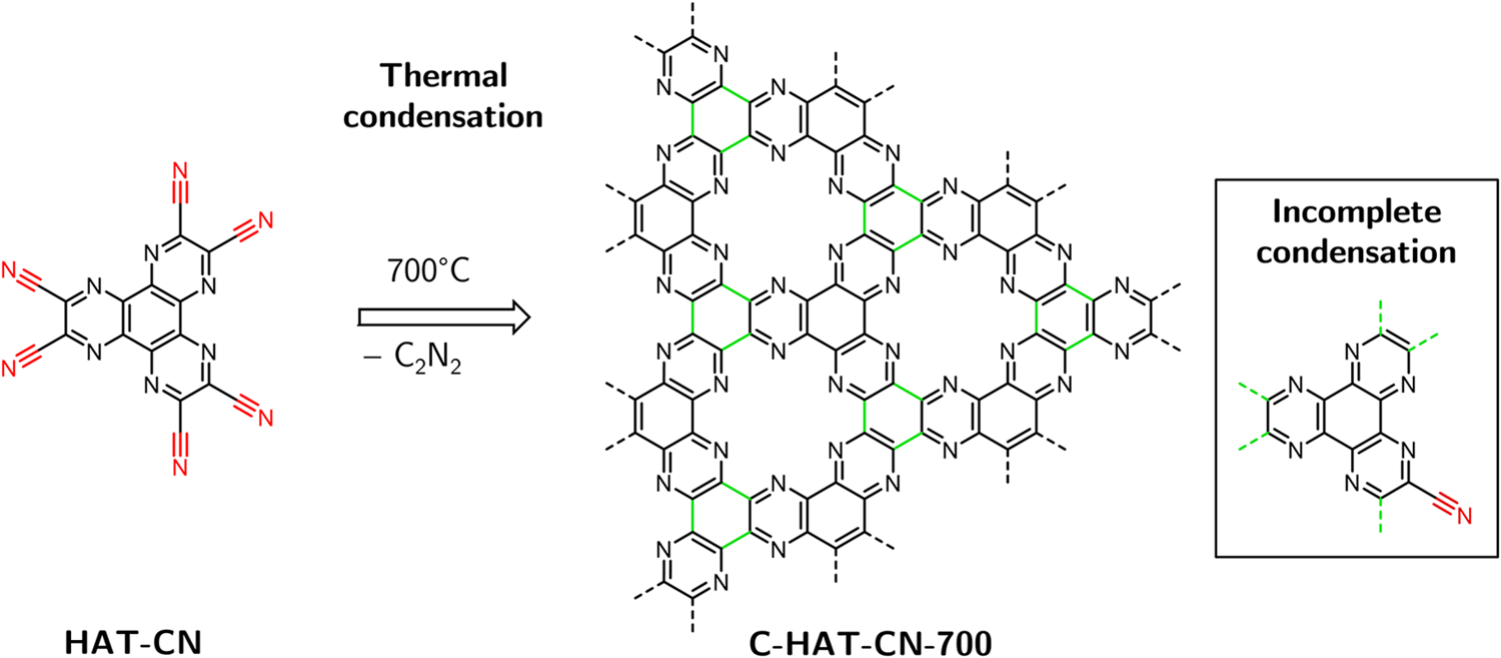
Synthesis of amorphous, carbonaceous C-HAT-CN-700 by thermal treatment of the HAT-CN precursor at 700 °C, leading to the C_2_N-framework with inherently bound nitrile groups due to incomplete condensation. Displayed according to Walczak et al.^39^.

Recently, a metal-free nitrogen-rich carbon-based electrocatalyst of C_2_N-stoichiometry was introduced, which shows promising electrocatalytic behavior for HER and the nitrogen reduction reaction (NRR).^38,39^ The synthesis strategy follows a simple fabrication approach by which the precursor hexaazatriphenylene hexacarbonitrile (HAT-CN) condenses via its nitrile functions by thermal treatment to form the amorphous and highly porous carbonaceous material C-HAT-CN-XX (XX is the condensation temperature in °C) with a porous and local 2D structure. Its properties such as the nitrogen content and the degree of condensation are controllable via the carbonization temperature. It was shown previously that the material synthesized at 700 °C (C-HAT-CN-700) contains numerous nitrile functionalities due to incomplete condensation of the precursor molecules, as is shown in Figure 1.^39^ As a result, this article presents an intriguing combination of two research fields: On the one hand, it is a promising electrocatalyst relevant for technological application; on the other hand, it is of interest in electrochemical Stark spectroscopy owing to the inherently bound nitrile groups.

In this work, we investigate the character of the CN groups bound at the C_2_N-framework of the metal-free carbon-based electrocatalyst C-HAT-CN-700 using both Raman spectroscopy and first-principle simulations. Furthermore, we use in situ electrochemical Raman spectroscopy to monitor the spectroscopic behavior of the ν(C≡N) peak under both noncatalytic and HER conditions, and we discuss possible processes and effects at the solid–liquid interface as origins of the observed frequential shifts during electrochemical measurements.

## Results and Discussion

**Figure 2:**
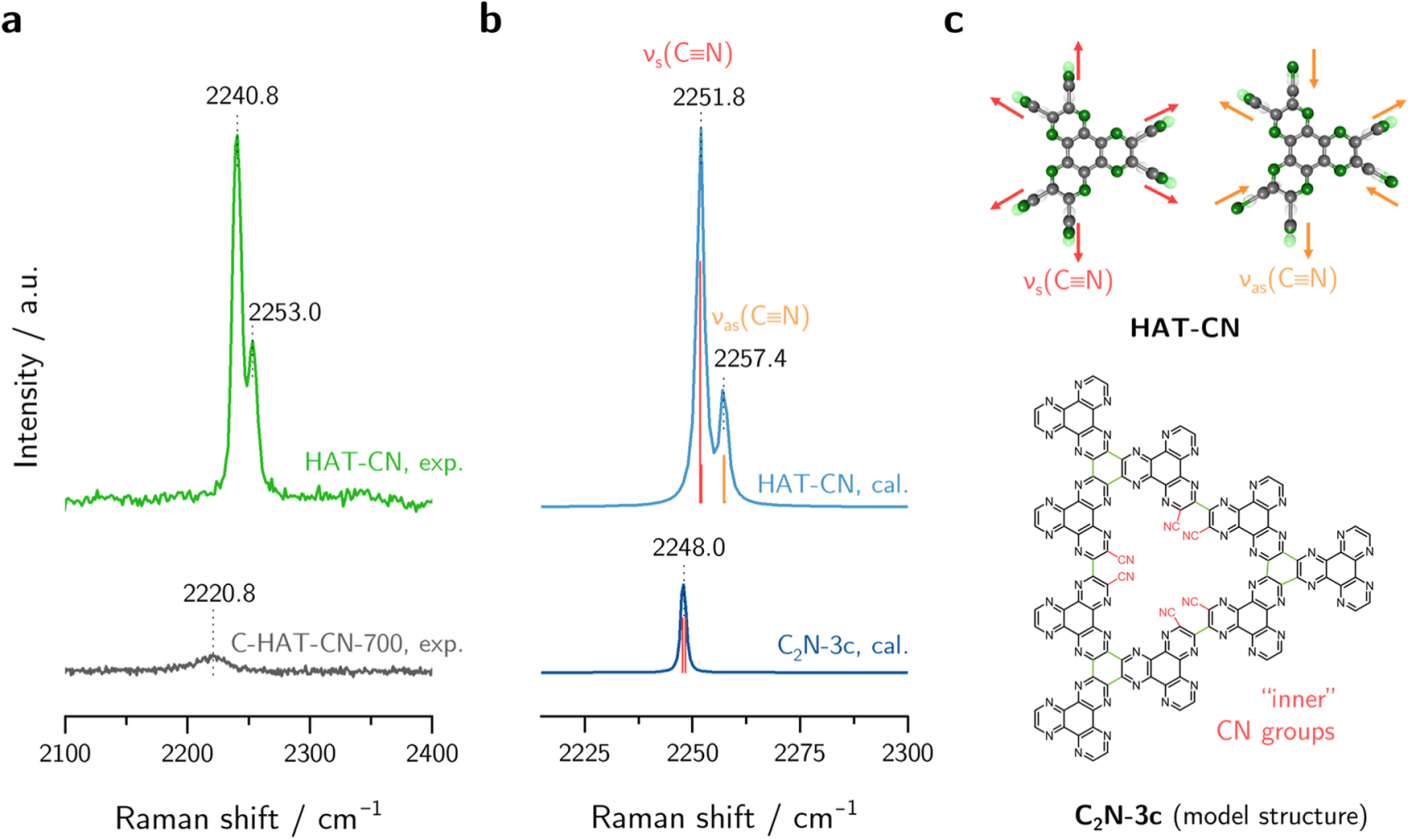
(a) Experimental Raman spectra of HAT-CN (top, 640 nm excitation wavelength) and C-HAT-CN-700 (bottom, 405 nm excitation wavelength), (b) calculated Raman spectra of HAT-CN (top) and calculated normal modes of C_2_N-3c model structure with “inner” CN groups, and (c) visualization of the symmetric, ν_s_(C≡N), and antisymmetric C≡N stretching vibrations, ν_as_(C≡N), in HAT-CN (top) and model structure of the condensed material C_2_N-3c (bottom).

Describing structural binding motifs in amorphous systems such as C-HAT-CN-XX is both challenging and essential for the accurate interpretation of spectral data. In order to understand the chemical environment of incorporated nitrile moieties that are of interest in electrochemical Stark spectroscopy, Raman spectra of the HAT-CN precursor and of the carbonized material C-HAT-CN-700 were recorded. The spectral window around 2250 cm^–1^, relevant for analyzing vibrational modes of the nitrile group, is shown in Figure 2a. The full experimental Raman and infrared (IR) spectra, including the in-plane vibrations of the C_2_N-framework between 1100 and 1700 cm^–1^, are shown in Figure S1.

The precursor material HAT-CN exhibits prominent bands with two resolved maxima at 2240.8 and 2253.3 cm^–1^, respectively, which are assigned to the stretching vibration ν(C≡N). DFT calculations reveal that the molecule has six different vibrational modes in the respective frequency range with a high contribution of the −C≡N bond stretching. The calculated Raman spectrum displaying the nitrile stretching vibrations is shown in Figure 2b and the full spectrum in Figure S2. The modes can be divided into two groups with slightly different frequencies described as symmetric and antisymmetric stretching, ν_s_(C≡N) and ν_as_(C≡N), depending on their relative phase toward each other. The two most intense vibrations are exemplified in Figure 2c.

The Raman spectra of the carbonized material C-HAT-CN-700 show a less intense peak in the same region that can be assigned to nitrile groups that are present in the material due to incomplete condensation when forming the expanded C_2_N-network. The peak maximum is shifted to lower wavenumbers by 20 cm^–1^ with respect to the ν_s_(C≡N) peak in the precursor molecule and is found at 2220.8 cm^–1^. In order to determine the nature of the nitrile groups within the amorphous material and interpret the experimental spectra, an apt structural description of the network is needed. The Raman spectra of a variety of model structures were calculated (see Figure S3). It was found that the structure size did not have a strong influence on the peak position when the nitrile functions are bound as terminal (“outer”) groups at the oligomers. However, when “defect sites” were included, which represent an incomplete condensation of the network leading to “inner” nitrile groups, their vibrational frequency was red-shifted by 3.8 cm^–1^ and 9.4 cm^–1^ compared to the calculated ν_s_(C≡N) and ν_as_(C≡N) modes of HAT-CN, respectively. Furthermore, both the symmetric and antisymmetric stretching modes of the inner nitrile groups have similar vibrational frequencies, around 2248.0 cm^–1^. These findings stand in good accordance with the experimental spectrum of C-HAT-CN-700 that shows only one broader peak, which can be well fitted with one band of Gaussian shape. Therefore, it is concluded that the observed ν(C≡N) peak can be assigned to several stretching modes of nitrile groups that have defect character and are embedded within the amorphous C_2_N-network.

**Figure 3:**
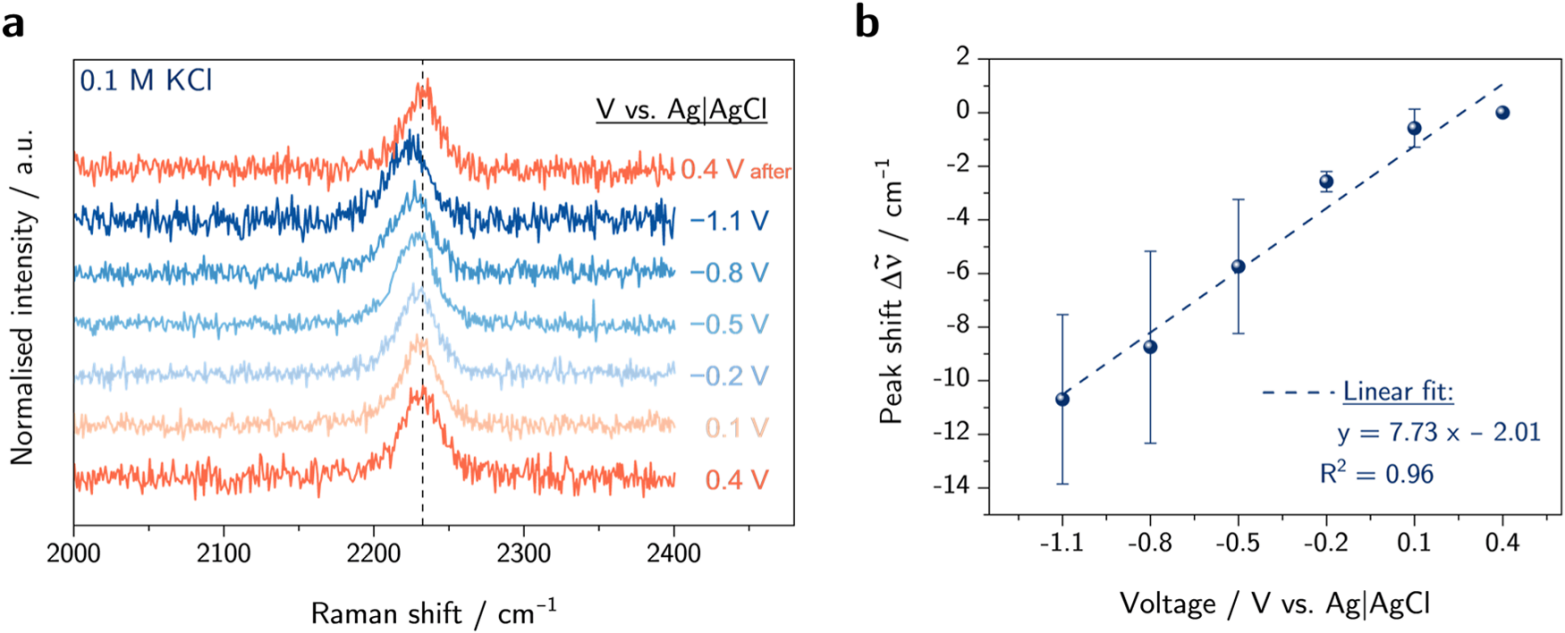
(a) Raman spectra of C-HAT-CN-700 in KCl electrolyte (0.1 M) under different applied potential and (b) ν(C≡N) peak shift Δ*ν̃* = *ν̃*–*ν̃*_0_ with respect to the initial band position *ν̃*_0_ at 0.4 V as a function of applied potential.

As a next step, electrochemical in situ Raman spectroscopy was carried out on C-HAT-CN-700, and the nitrile stretching vibration was investigated by varying the applied potential in the KCl electrolyte (0.1 M), as shown in Figure 3. When lowering the applied potential, the ν(C≡N) band position shifts toward lower wavenumbers, resulting in a red-shift by in total (−11 ± 3) cm^–1^ over the measured potential range of 1.5 V. Afterwards, the spectra at 0.4 V vs Ag|AgCl were measured again. It is observed that the peak is located again near the original wavenumber at (−2.0 ± 2.6) cm^–1^, which proves the reversibility of the process. By plotting the peak shift vs applied voltage, a linear trend is obtained with a slope of 7.73 cm^–1^ V^–1^. The corresponding response currents are listed in Figure S4. In comparison with literature, similar slope values between 5.1 and 8.0 cm^–1^ V^–1^ are found for electrochemical systems based on 4-mercaptobenzonitrile with CN reporter groups.^17,30,31^ Interestingly, the obtained slope here not only shows the same sign but also its magnitude is in good accordance with literature values. Interpreting the observed frequency shift as a Stark shift raises intriguing considerations, which will be discussed further in the following.

**Figure 4:**
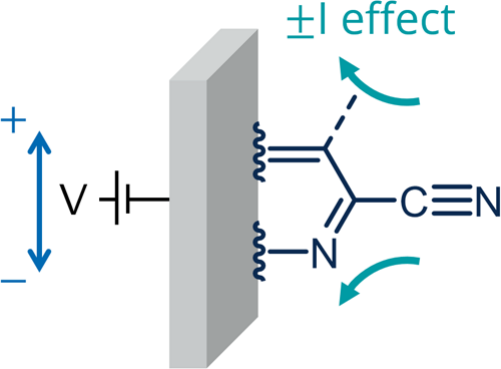
Schematic illustration of the possible inductive effect (±I effect) of the electrode on the CN functional group.

First, a linear Stark shift is generally observed as the scalar product of the vector of the local electric field and the difference dipole moment that is directed along the CN bonding axis according to eq 1. By approximating the short-ranged local electric field as perpendicular to the plane of the extended C_2_N-framework, an orientation of the CN groups out-of-plane is required in order to receive a non-zero scalar product and therefore to observe Stark shifts. Considering the calculated model structures, it is found that neighboring “inner” nitrile functionalities display a dihedral angle of >90° due to sterical hindrance, forming a nonplanar system as shown in Figure S5. In addition, “outer” CN groups display dihedral angles of up to 71°. The chemical structure of the investigated system therefore meets the geometrical prerequisite for a measurable VSE. To continue with this, the theory of Smith and White,^40^ with extension by Hildebrand et al.,^28,30^ allows the conversion of the experimentally obtained slope into the Stark tuning rate that describes the band shift per change in electric field strength (unit: cm^–1^ per V cm^–2^). However, this approach requires atomically precise knowledge of the chemical environment around the Stark reporter groups. Therefore, the frequency-to-field calibration is not straightforward for structurally less defined systems such as amorphous C-HAT-CN-700, and further investigations in this direction are beyond the scope of this work. Second, local electric fields are strongly influenced by the composition of the ECDL that builds the solvation field at the electrode surface in accordance with the applied potential. In order to check the influence of the counterions within the ECDL, the peak position of the CN groups is investigated depending on the ionic strength of the KCl electrolyte. As shown in Figure S6, no peak shifts are observed in different ionic strengths at the same applied potential. Thus, the impact of the solvation field does not seem to be the cause of the observed peak shift during electrochemical measurement. Third, electroinductive effects (“through-bond effects”, conceptually depicted in Figure 4) are likely to have a non-negligible influence on the peak position in addition to electrostatic effects. It remains a widely discussed question to what extent these two effects contribute to observed peak shifts in electrochemical Stark spectroscopy, and there is indication in literature that the electrode’s inductive effects seem to contribute to a larger percentage to observed frequency shifts at fully conjugated systems.^31,33^ Since the CN groups in the here investigated system are directly bond to a conjugated system and the magnitude of the slope of 7.73 cm^–1^ V^–1^ is rather large compared to literature values obtained for aliphatic systems,^28^ it can be concluded that a combination of both electrostatic and electroinductive effects is expected to cause the observed red-shift.

**Figure 5:**
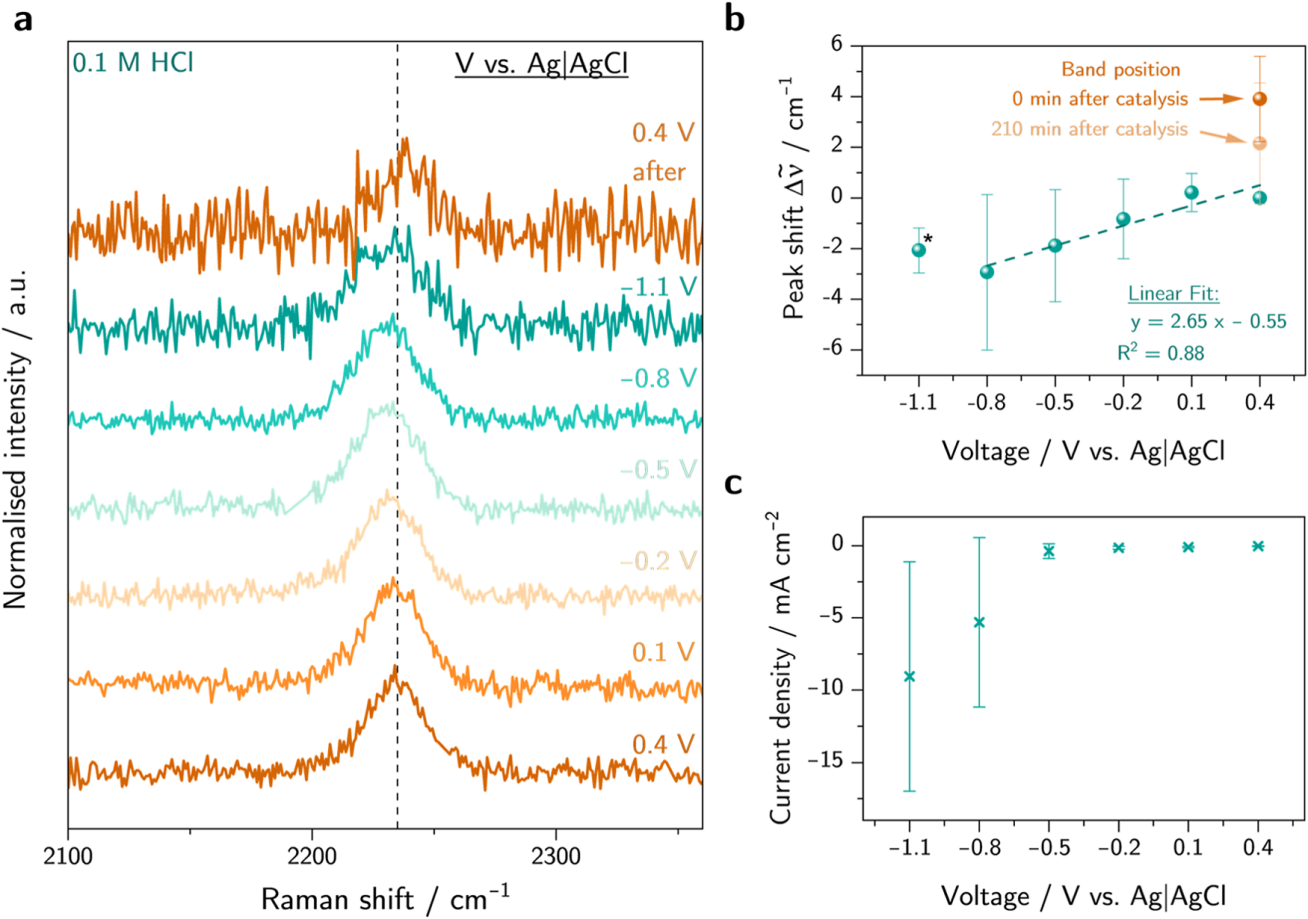
(a) Raman spectra of C-HAT-CN-700 in HCl electrolyte (0.1 M) under different applied potential, (b) position of the ν(C≡N) band maximum as a function of applied potential (blue) and after catalysis (orange), and (c) average current densities recorded at each potential. *Only two spectra were measured at −1.1 V due to strong gas evolution and a loss of spectral quality.

C-HAT-CN-700 was found to promote HER in the HCl electrolyte (0.1 M), and the respective linear sweep voltammetry (LSV) curve is shown in Figure S7. These findings are in line with previous results by Zhang et al. on the electrocatalytic performance of this type of material.^38^ Exchanging the electrolyte from KCl to HCl changes the electrochemical properties of the system under negative applied potential since the much lower pH value favors the HER. Analogue in situ Raman measurements are conducted in HCl electrolyte, and the results are shown in Figure 5. Note that the decreasing spectral quality under a high negative potential and when going back to the starting potential is due to H_2_ gas bubbles that prevent good laser focus from the Raman microscope. Additionally, the measured currents at the same applied potential vary strongly between experiments, as shown in Figure 5c, which correlates with the large standard deviation in Raman peak shifts at lower potentials.

The obtained frequency shifts no longer display a linear dependency over the full potential range between 0.4 and −1.1 V. A linear shift in peak position is present for potentials from 0.4 to −0.8 V where no catalytic currents are observed albeit with a lower slope of 2.65 cm^–1^ V^–1^ compared to that determined in KCl electrolyte. Below −0.8 V, the catalytic current increases, and the peak does not red-shift further. A possible explanation for the lower slope within the precatalytic region could be found in the exchange of K^+^ to H^+^. The presence of H^+^ ions could possibly lead to potential-induced, successive protonations of the pyrazinic nitrogen at the C_2_N-framework, and consequently, the previously discussed electroinductive effect of the electrode is weakened leading to an overall lower peak shift of the ν(C≡N) mode. At the catalytic regime below – 0.8 V, the largely increased current density due to faradaic processes could compensate for both an electron-pushing effect of the electrode and the higher electric field caused by the more negative applied potential due to a higher ohmic drop. A similar trend has been reported in the literature where constant peak positions are observed under electrocatalytic conditions.^29,36^

**Figure 6:**
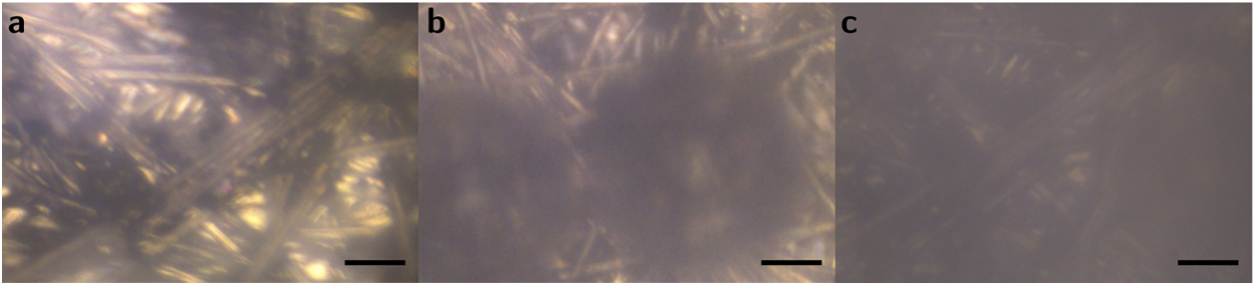
Microscope image of C-HAT-CN-700 (black dots) on carbon paper support electrodes (light gray strands), (a) in HCl electrolyte (0.1 M) at 0.4 V, (b) in HCl (0.1 M) at – 1.1 V during HER (bubble formation); (c) in HCl at 0.4 V after the measurement where the image is very blurred possibly due to a film of H_2_ nano- and microbubbles attached to the electrode. Scale bar = 20 μm.

Interestingly, when measuring again at the initial potential of 0.4 V after catalysis, a blue-shift by (3.9 ± 1.7) cm^–1^ toward higher wavenumbers is observed with good reproducibility. The same frequency shift was observed when the system was kept at a constant potential of −1.1 V for 2 h, and afterward, the starting potential was measured again as shown in Figure S8. The peak shift is semipermanent and seems to red-shift back toward its original position over an observed time scale of up to 4 h (see Figure S9). No shift was observed when the potential did not reach HER conditions; therefore, it can be concluded that these two events (hydrogen evolution and peak shift) are correlated, indicating that the catalytic process leads to a conditioning of the electrode, which manifests in a temporary ν(C≡N) blue-shift. Both the magnitude of the shift and the time dependence of the back-shift varied strongly for each experiment. However, the peak position and relative intensity measured at the dry catalyst material before and after catalysis remain unchanged (Figure S10). Several reasons for the observed phenomenon are plausible and are discussed in the following:

First, the observed shift could originate from structural changes of the catalyst during HER. Zhang et al. proposed a structural change that includes the electrochemical removal of the CN groups leaving radicals at the elimination position.^38^ They assumed a reduction of nitrogen content by 5.4–6.6% due to the electrochemical etching of CN groups resulting from the activation of the materials during electrochemical cycling over a wide voltage range prior to catalytic turnover. However, under the conditions chosen in the present investigations, a comparison of the normalized Raman spectra of the dry catalyst material before and after the electrochemical treatment (shown in Figure S10) did not show any significant change. The signal intensity of the CN peak of the dry material at 2229 cm^–1^ does not change before and after the treatment, so the removal of nitrile groups cannot act as an explanation under the conditions of the present spectroscopic measurement. Furthermore, the reversibility of the peak shift back to the initial position over time cannot be adequately explained by a permanent structural change such as the complete elimination of a functional group. Alternatively, the blue-shift could be explained following the previously mentioned argumentation regarding electrochemical Stark spectroscopy. However, the influence of electroinductive effects can be ruled out in this case because the ν(C≡N) peak before and after catalysis is recorded at the same applied potential of 0.4 V. It is therefore very likely that the origin of the observed blue-shift after HER-catalysis can be primarily attributed to electrostatic effects, as described by the VSE in eq 1. Specifically, a restructuring of the ECDL during catalysis and the resulting change in noncovalent interactions affect the local electric field environment at the interfacial layer and are monitored by the Stark shift of the nitrile groups as reporter groups. From a mechanistic point of view, the electrochemical conversion process can be split into different steps, each having an impact on electric fields and vice versa. Presumably, the consumption of H^+^ during the reaction generates a proton concentration gradient through the ECDL. To verify this assumption, the influence of the H^+^ concentration on the CN peak position was measured over a large HCl concentration range (Figure S11). Although no clear trend is apparent, there could be a small correlation between a lower H^+^ concentration and an observed red-shift. Consequently, the H^+^ consumption during the HER should be considered as an additional influencing factor for the peak shifts observed at lower applied potential. However, since the opposite peak shift is observed after the measurement, a possible H^+^ depletion in the ECDL due to the HER does not seem to provide a conclusive explanation for the observed blue-shift. In addition, proton mass transport via the Grotthuß mechanism is considered to be very fast, so a concentration gradient due to HER is assumed to vanish quicker than the time scale over which the back-shift after the measurement is observed. Finally, the production of H_2_ at the electrode surface also influences the composition of the interface. The production of gaseous H_2_ is observed in the microscopic images during catalysis (see Figure 6). Some bubbles are still visible in the microscopic image after catalysis, and the image remains furthermore blurry, which could be due to nonvisible nano- and microbubbles. The process of H_2_ evolution, starting from the formation of the surface-bound diatomic hydrogen molecule by faradaic processes, the formation of nano- and microbubbles, until the detachment of gaseous H_2_ from the electrode, and the influence of these (uncharged!) H_2_ species on the local electric field is not widely understood yet. However, they could be the origin of the blue-shift of the experiment. The time scale as well as the high standard deviation of the ν(C≡N) peak positions at different points in time after the measurement would support this hypothesis because the H_2_ detachment from a highly porous electrode surface is considered to be a rather random process.

## Conclusions

In this work, we studied the nitrogen-rich carbon material C-HAT-CN-700 that displays electrocatalytic activity for HER in the context of electrochemical Stark spectroscopy. Supported by experimental and calculated Raman spectra, we concluded that inherently bound nitrile groups display a defect character, as they are bound as “inner” groups within the C_2_N-framework. Using electrochemical in situ Raman spectroscopy in KCl electrolyte, it was demonstrated that these groups act as molecular sensor groups displaying a linear dependency between applied potential and peak shift of the stretching modes ν(C≡N) found at ca. 2230 cm^–1^. Both the influence of the local electric field in the classic interpretation of the observation as a Stark shift and the role of the electrode as an electron donor displaying an inductive effect were discussed with the conclusion that both effects are likely to contribute to the observed frequency shift. Yet, clear disentanglement of both contributions is not achieved at this point. Further in-depth knowledge of the bonding structure within the amorphous system and the exact composition of the heterogeneous interface on a subnanometer scale would be required here. When changing electrolyte to HCl in order to favor HER, the CN sensor groups indicated an alteration of the heterogeneous interface. Here, the linear dependency is decreased at precatalytic conditions, possibly due to potential-induced protonation that can be considered as the first crucial step within the HER-catalytic cycle and requires multiple Lewis-basic sites to be present at the active centers. Additionally, a semipermanent reverse peak shift (blue-shift) is observed after the HER-catalysis that indicates a temporary conditioning of the electrode due to the catalytic turnover. We discussed several mechanistic influences as possible origins of this effect. The modulation of the electric field environment at the interfacial region, presumably due to noncovalent interaction with dissolved or gaseous H_2_, was found to fit the time scale as well as the high variance of the observed phenomenon.

Overall, the electrochemical Stark shift study on the metal-free and amorphous C-HAT-CN-700 electrocatalyst with nitrile sensor groups gives intriguing insight into the complexity of the mechanism behind an electrocatalytic process and can be used to identify changes at the electrode–electrolyte interface prior to catalytic turnover. Moreover, to give a perspective, multiple factors such as nonspecific interactions, mass transport phenomena, gas evolution, and time-dependent changes within the system need to be considered for a high-performing and technologically relevant system that go beyond the classic kinetic cycle based on covalent bonding and intermediate formation.

## Materials and Methods

### Material Synthesis

All chemicals were used as received. HAT-CN as well as HAT-CN-700 were synthesized according to previously reported procedures.^38,41^ In short, C-HAT-CN-700 was synthesized using 200 mg of HAT-CN for carbonization in a horizontal tubular furnace at 700 °C for 1 h under Ar gas flow. The heating ramp was set at 2 °C min^–1^.

### Electrochemical Measurements

Electrode preparation was conducted following the protocol by Zhang et al.^38^ In short, a catalyst ink was prepared by dispersing 1 mg of C-HAT-CN-700 in 100 μL of absolute ethanol (>99.8%, purchased from Fisher Chemical) and 20 μL of Nafion dispersion (5 w/w% in water and 1-propanol, purchased from Thermo Fisher Scientific), followed by sonification for 30 min. The blank sample was prepared without the C-HAT-CN-700 material. The ink was drop-casted onto a carbon-paper support electrode (Toray Carbon Paper, purchased from Thermo Fisher Scientific) with a diameter of 1.2 cm and dried at 80 °C for 4 h.

Electrochemical measurements were carried out at a Vertex.One potentiostat (Ivium Technologies) in a three-electrode setup. KCl (Grüssing GmbH) and HCl (VWR Chemicals) electrolytes were prepared using Milli-Q water (<0.055 μS cm^–1^). A Pt-wire was used as the counter electrode, and all potentials given in this work were measured against an Ag|AgCl|KCl (3 M) reference electrode. The electrolyte was purged with argon gas for 15 min prior to the measurements, and linear sweep voltammetry (LSV) curves were conducted with continuous gas flow. Current densities were calculated from the response currents divided by the nominal (geometrical) surface area of the electrode that is exposed to the electrolyte and equals 0.64 cm^2^.

### Raman Spectroscopy

Raman spectroscopical measurements were performed at a confocal Raman spectrometer (S&I Monovista CRS+) with a liquid nitrogen-cooled detector. If not stated otherwise, an excitation wavelength of 405 nm (Toptica Top mode diode laser) at 2.748 mW and 1500 g/mm holographic grating was used. A 640 nm laser (LASOS diode pumped solid state laser) was used for HAT-CN spectra due to high fluorescence background. The laser was focused onto the sample using a Nikon 20x objective (0.35 NA, 20 mm WD) and an *x*-*y*-stage (Scan IM 200 × 200, Märzhäuser GmbH & Co. KG, Germany). In situ spectra were received by chronoamperometric measurements with applied potentials from 0.4 to −1.1 V in steps of 0.3 V and 4 × 180 s accumulation time. The experiment was repeated 3 times in KCl (0.1 M) and 4 times in HCl (0.1 M). Only three spectra could be measured at −0.8 V and two spectra at −1.1 V due to loss of spectral quality due to the gas evolution. All Raman spectra shown are baseline-corrected, calibrated with respect to acetonitrile and toluene reference Raman spectra. Normalization is carried out in relation to the highest peak intensity.

### Attenuated Total Reflectance Fourier Transform Infrared (ATR-FTIR) Spectroscopy

FTIR spectra were recorded with a Bruker Tensor II instrument in ATR mode using a diamond crystal and Pt stamp. A total of 256 scans were accumulated with a resolution of 2 cm^–1^. Background spectra were subtracted, and absorbance spectra were gained with automatic ATR correction.

### DFT Calculations

The normal-mode frequencies for all six molecules were obtained via normal-mode analysis using the FHI-aims package.^42^ We used the settings that we previously identified as optimal for carbon-based materials^43^ and which we briefly summarize here. For the DFT calculations, we used the Perdew–Burke–Ernzerhof (PBE) exchange-correlation functional^44^ in combination with the Tkatchenko–Scheffler dispersion correction.^45^ Numerical atom-centered orbitals of *tier2* quality were used together with “tight” numerical settings.^42^ After a tight geometry optimization, *6N* structures were formed by shifting each atom *N* by 0.001 Å in the ± *x*, ± *y*, and ± *z* directions. The DFT forces were calculated for each of these shifted structures to construct the mass-weighted Hessian matrix, which was evaluated numerically via central finite differences. The obtained normal modes for all six molecules are shown with blue bars in Figure S3.

To confirm that the symmetric (ν_s_(C≡N)) and antisymmetric (ν_as_(C≡N)) C≡N stretching vibrations at ≅2251 cm^–1^ and ≅2257 cm^–1^ are Raman active, we computed the Raman intensities for the molecules HAT-CN, C_2_N-1, and C_2_N-2. The Raman spectra are shown in Figure S3 (solid red lines). The polarizability tensors are calculated for each of the *6N* displaced structures via density functional perturbation theory (DFPT)^46−48^ as implemented in the CP2K program package.^49^ For the DFPT calculations, we employ the PBE functional^45^ in combination with Goedecker–Teter–Hutter pseudopotentials^50−52^ and the DZVP-MOLOPT-SR-GTH basis sets.^53^ For each normal mode, the Raman intensity was computed from the first derivative of the polarizabilities with respect to mass-weighted normal mode displacements, employing a central finite difference scheme. The unpolarized (total) intensities are reported for an incident laser wavelength of 405 nm, and the final spectra were broadened using Lorentzian broadening. For detailed information on the generation of normal-mode frequencies and Raman intensities, please refer to ref (43). All input and output files of the calculations are available on Zenodo database.^54^

## Supporting Information Available

Additional Raman and ATR-FTIR spectra; CN dihedral angles obtained for model structure; additional electrochemical measurements; ν(C≡N) at different KCl and HCl concentrations; ν(C≡N) during chronoamperometry in HCl for 2h; ν(C≡N) peak shift after catalysis; additional references (PDF)

## References

[ref1] Fried S. D.; Boxer S. G. Measuring Electric Fields and Noncovalent Interactions Using the Vibrational Stark Effect. Acc. Chem. Res. 2015, 48 (4), 998–1006. 10.1021/ar500464j. 25799082 PMC4667952

[ref2] Fried S. D.; Boxer S. G. Electric Fields and Enzyme Catalysis. Annu. Rev. Biochem. 2017, 86, 387–415. 10.1146/annurev-biochem-061516-044432. 28375745 PMC5600505

[ref3] Fried S. D.; Bagchi S.; Boxer S. G. Extreme Electric Fields Power Catalysis in the Active Site of Ketosteroid Isomerase. Science 2014, 346 (6216), 1510–1514. 10.1126/science.1259802. 25525245 PMC4668018

[ref4] Warshel A. Electrostatic Basis of Structure-Function Correlation in Proteins. Acc. Chem. Res. 1981, 14 (9), 284–290. 10.1021/ar00069a004.

[ref5] Pocker Y.; Buchholz R. F. Electrostatic Catalysis of Ionic Aggregates. I. Ionization and Dissociation of Trityl Chloride and Hydrogen Chloride in Lithium Perchlorate-Diethyl Ether Solutions. J. Am. Chem. Soc. 1970, 92 (7), 2075–2084. 10.1021/ja00710a047.

[ref6] Abraham M. H. Substitution at Saturated Carbon. Part XIV. Solvent Effects on the Free Energies of Ions, Ion-Pairs, Non-Electrolytes, and Transition States in some SN and SE Reactions. J. Chem. Soc., Perkin Trans. 2 1972, (10), 1343–1357. 10.1039/p29720001343.

[ref7] Yang C.; Liu Z.; Li Y.; Zhou S.; Lu C.; Guo Y.; Ramirez M.; Zhang Q.; Li Y.; Liu Z.; Houk K. N.; Zhang D.; Guo X. Electric Field–Catalyzed Single-Molecule Diels-Alder Reaction Dynamics. Sci. Adv. 2021, 7 (4), eabf0689 10.1126/sciadv.abf0689. 33523936 PMC7817103

[ref8] Aragonès A. C.; Haworth N. L.; Darwish N.; Ciampi S.; Bloomfield N. J.; Wallace G. G.; Diez-Perez I.; Coote M. L. Electrostatic Catalysis of a Diels–Alder Reaction. Nature 2016, 531 (7592), 88–91. 10.1038/nature16989. 26935697

[ref9] Joy J.; Stuyver T.; Shaik S. Oriented External Electric Fields and Ionic Additives Elicit Catalysis and Mechanistic Crossover in Oxidative Addition Reactions. J. Am. Chem. Soc. 2020, 142 (8), 3836–3850. 10.1021/jacs.9b11507. 31994390

[ref10] Shaik S.; Danovich D.; Joy J.; Wang Z.; Stuyver T. Electric-Field Mediated Chemistry: Uncovering and Exploiting the Potential of (Oriented) Electric Fields to Exert Chemical Catalysis and Reaction Control. J. Am. Chem. Soc. 2020, 142 (29), 12551–12562. 10.1021/jacs.0c05128. 32551571

[ref11] Weaver M. J. Electrostatic-Field Effects on Adsorbate Bonding and Structure at Metal Surfaces: Parallels between Electrochemical and Vacuum Systems. Appl. Surf. Sci. 1993, 67 (1), 147–159. 10.1016/0169-4332(93)90307-W.

[ref12] Karlberg G. S.; Rossmeisl J.; Nørskov J. K. Estimations of Electric Field Effects on the Oxygen Reduction Reaction Based on the Density Functional Theory. Phys. Chem. Chem. Phys. 2007, 9 (37), 5158–5161. 10.1039/b705938h. 17878993

[ref13] Ramuglia A. R.; Budhija V.; Ly K. H.; Schwalbe M.; Weidinger I. M. Proton Activation in the Presence of a Weak Acid Facilitated by Second Coordination Sphere Effects in Iron Porphyrins. ChemElectroChem. 2023, 10 (23), e202300369 10.1002/celc.202300369.

[ref14] Li H.; Wei P.; Gao D.; Wang G. In Situ Raman Spectroscopy Studies for Electrochemical CO_2_ Reduction over Cu Catalysts. Curr. Opin. Green Sustainable Chem. 2022, 34, 100589 10.1016/j.cogsc.2022.100589.

[ref15] Borrelli M.; An Y.; Querebillo C. J.; Morag A.; Neumann C.; Turchanin A.; Sun H.; Kuc A.; Weidinger I. M.; Feng X. Donor-Acceptor Conjugated Acetylenic Polymers for High-Performance Bifunctional Photoelectrodes. ChemSusChem 2024, 17 (7), e202301170 10.1002/cssc.202301170. 38062976

[ref16] Weaver J. B.; Kozuch J.; Kirsh J. M.; Boxer S. G. Nitrile Infrared Intensities Characterize Electric Fields and Hydrogen Bonding in Protic, Aprotic, and Protein Environments. J. Am. Chem. Soc. 2022, 144 (17), 7562–7567. 10.1021/jacs.2c00675. 35467853 PMC10082610

[ref17] Wright D.; Sangtarash S.; Mueller N. S.; Lin Q.; Sadeghi H.; Baumberg J. J. Vibrational Stark Effects: Ionic Influence on Local Fields. J. Phys. Chem. Lett. 2022, 13 (22), 4905–4911. 10.1021/acs.jpclett.2c01048. 35623089 PMC9189927

[ref18] Che F.; Gray J. T.; Ha S.; Kruse N.; Scott S. L.; McEwen J.-S. Elucidating the Roles of Electric Fields in Catalysis: A Perspective. ACS Catal. 2018, 8 (6), 5153–5174. 10.1021/acscatal.7b02899.

[ref19] Sellner B.; Valiev M.; Kathmann S. M. Charge and Electric Field Fluctuations in Aqueous NaCl Electrolytes. J. Phys. Chem. B 2013, 117 (37), 10869–10882. 10.1021/jp405578w. 23906325

[ref20] Pacchioni G.; Lomas J. R.; Illas F. Electric Field Effects in Heterogeneous Catalysis. J. Mol. Catal. A: Chem. 1997, 119 (1), 263–273. 10.1016/S1381-1169(96)00490-6.

[ref21] Chen L. D.; Urushihara M.; Chan K.; Nørskov J. K. Electric Field Effects in Electrochemical CO_2_ Reduction. ACS Catal. 2016, 6 (10), 7133–7139. 10.1021/acscatal.6b02299.

[ref22] Liu M.; Pang Y.; Zhang B.; Luna P. de.; Voznyy O.; Xu J.; Zheng X.; Dinh C. T.; Fan F.; Cao C.; Arquer F. P. G. de.; Safaei T. S.; Mepham A.; Klinkova A.; Kumacheva E.; Filleter T.; Sinton D.; Kelley S. O.; Sargent E. H. Enhanced Electrocatalytic CO_2_ Reduction Via Field-Induced Reagent Concentration. Nature 2016, 537 (7620), 382–386. 10.1038/nature19060. 27487220

[ref23] Xu J.; Xue X.-X.; Shao G.; Jing C.; Dai S.; He K.; Jia P.; Wang S.; Yuan Y.; Luo J.; Lu J. Atomic-Level Polarization in Electric Fields of Defects for Electrocatalysis. Nat. Commun. 2023, 14 (1), 7849 10.1038/s41467-023-43689-y. 38030621 PMC10686988

[ref24] Ling F.; Liu X.; Jing H.; Chen Y.; Zeng W.; Zhang Y.; Kang W.; Liu J.; Fang L.; Zhou M. Optimizing Edges and Defects of Supported MoS_2_ Catalysts for Hydrogen Evolution Via an External Electric Field. Phys. Chem. Chem. Phys. 2018, 20 (41), 26083–26090. 10.1039/C8CP03407A. 30109330

[ref25] Yan M.; Pan X.; Wang P.; Chen F.; He L.; Jiang G.; Wang J.; Liu J. Z.; Xu X.; Liao X.; Yang J.; Mai L. Field-Effect Tuned Adsorption Dynamics of VSe_2_ Nanosheets for Enhanced Hydrogen Evolution Reaction. Nano Lett. 2017, 17 (7), 4109–4115. 10.1021/acs.nanolett.7b00855. 28585826

[ref26] Bhattacharyya D.; Videla P. E.; Palasz J. M.; Tangen I.; Meng J.; Kubiak C. P.; Batista V. S.; Lian T. Sub-Nanometer Mapping of the Interfacial Electric Field Profile Using a Vibrational Stark Shift Ruler. J. Am. Chem. Soc. 2022, 144 (31), 14330–14338. 10.1021/jacs.2c05563. 35905473

[ref27] Bublitz G. U.; Boxer S. G. STARK SPECTROSCOPY: Applications in Chemistry, Biology, and Materials Science. Annu. Rev. Phys. Chem. 1997, 48 (1), 213–242. 10.1146/annurev.physchem.48.1.213. 9348658

[ref28] Staffa J. K.; Lorenz L.; Stolarski M.; Murgida D. H.; Zebger I.; Utesch T.; Kozuch J.; Hildebrandt P. Determination of the Local Electric Field at Au/SAM Interfaces Using the Vibrational Stark Effect. J. Phys. Chem. C 2017, 121 (40), 22274–22285. 10.1021/acs.jpcc.7b08434.

[ref29] Shi H.; Cai Z.; Patrow J.; Zhao B.; Wang Y.; Wang Y.; Benderskii A.; Dawlaty J.; Cronin S. B. Monitoring Local Electric Fields at Electrode Surfaces Using Surface Enhanced Raman Scattering-Based Stark-Shift Spectroscopy during Hydrogen Evolution Reactions. ACS Appl. Mater. Interfaces 2018, 10 (39), 33678–33683. 10.1021/acsami.8b11961. 30187745

[ref30] Schkolnik G.; Salewski J.; Millo D.; Zebger I.; Franzen S.; Hildebrandt P. Vibrational Stark Effect of the Electric-Field Reporter 4-Mercaptobenzonitrile as a Tool for Investigating Electrostatics at Electrode/SAM/Solution Interfaces. Int. J. Mol. Sci. 2012, 13 (6), 7466–7482. 10.3390/ijms13067466. 22837705 PMC3397537

[ref31] Lake W. R.; Meng J.; Dawlaty J. M.; Lian T.; Hammes-Schiffer S. Electro-inductive Effect Dominates Vibrational Frequency Shifts of Conjugated Probes on Gold Electrodes. J. Am. Chem. Soc. 2023, 145 (41), 22548–22554. 10.1021/jacs.3c07489. 37795975

[ref32] Sarkar S.; Patrow J. G.; Voegtle M. J.; Pennathur A. K.; Dawlaty J. M. Electrodes as Polarizing Functional Groups: Correlation between Hammett Parameters and Electrochemical Polarization. J. Phys. Chem. C 2019, 123 (8), 4926–4937. 10.1021/acs.jpcc.8b12058.

[ref33] Lake W. R.; Meng J.; Dawlaty J. M.; Lian T.; Hammes-Schiffer S. Electro-inductive Effects and Molecular Polarizability for Vibrational Probes on Electrode Surfaces. J. Phys. Chem. Lett. 2024, 15 (35), 9100–9104. 10.1021/acs.jpclett.4c02183. 39197102

[ref34] Rebstock J. A.; Zhu Q.; Baker L. R. Exploring the Influence of Interfacial Solvation on Electrochemical CO_2_ Reduction Using Plasmon-Enhanced Vibrational Sum Frequency Generation Spectroscopy. ChemCatChem. 2024, 16 (14), e202301301 10.1002/cctc.202301301.

[ref35] Ge A.; Videla P. E.; Lee G. L.; Rudshteyn B.; Song J.; Kubiak C. P.; Batista V. S.; Lian T. Interfacial Structure and Electric Field Probed by in Situ Electrochemical Vibrational Stark Effect Spectroscopy and Computational Modeling. J. Phys. Chem. C 2017, 121 (34), 18674–18682. 10.1021/acs.jpcc.7b05563.

[ref36] Patrow J. G.; Sorenson S. A.; Dawlaty J. M. Direct Spectroscopic Measurement of Interfacial Electric Fields near an Electrode under Polarizing or Current-Carrying Conditions. J. Phys. Chem. C 2017, 121 (21), 11585–11592. 10.1021/acs.jpcc.7b03134.

[ref37] Clark M. L.; Ge A.; Videla P. E.; Rudshteyn B.; Miller C. J.; Song J.; Batista V. S.; Lian T.; Kubiak C. P. CO_2_ Reduction Catalysts on Gold Electrode Surfaces Influenced by Large Electric Fields. J. Am. Chem. Soc. 2018, 140 (50), 17643–17655. 10.1021/jacs.8b09852. 30468391

[ref38] Zhang W.; Zhan S.; Qin Q.; Heil T.; Liu X.; Hwang J.; Ferber T. H.; Hofmann J. P.; Oschatz M. Electrochemical Generation of Catalytically Active Edge Sites in C_2_N-Type Carbon Materials for Artificial Nitrogen Fixation. Small 2022, 18 (42), 2204116 10.1002/smll.202204116. 36114151

[ref39] Walczak R.; Kurpil B.; Savateev A.; Heil T.; Schmidt J.; Qin Q.; Antonietti M.; Oschatz M. Template- and Metal-Free Synthesis of Nitrogen-Rich Nanoporous “Noble” Carbon Materials by Direct Pyrolysis of a Preorganized Hexaazatriphenylene Precursor. Angew. Chem., Int. Ed. 2018, 57 (33), 10765–10770. 10.1002/anie.201804359. 29882376

[ref40] Smith C. P.; White H. S. Theory of the Interfacial Potential Distribution and Reversible Voltammetric Response of Electrodes Coated with Electroactive Molecular Films. Anal. Chem. 1992, 64 (20), 2398–2405. 10.1021/ac00044a017. 19827817

[ref41] Kurpil B.; Savateev A.; Papaefthimiou V.; Zafeiratos S.; Heil T.; Özenler S.; Dontsova D.; Antonietti M. Hexaazatriphenylene Doped Carbon Nitrides—Biomimetic Photocatalyst with Superior Oxidation Power. Appl. Catal. B: Environ. 2017, 217, 622–628. 10.1016/j.apcatb.2017.06.036.

[ref42] Blum V.; Gehrke R.; Hanke F.; Havu P.; Havu V.; Ren X.; Reuter K.; Scheffler M. Ab Initio Molecular Simulations with Numeric Atom-Centered Orbitals. Comput. Phys. Commun. 2009, 180 (11), 2175–2196. 10.1016/j.cpc.2009.06.022.

[ref43] Bas E. E.; Garcia Alvarez K. M.; Schneemann A.; Heine T.; Golze D. Robust Computation and Analysis of Vibrational Spectra of Layered Framework Materials Including Host-Guest Interactions. J. Chem. Theory Comput. 2024, 20 (21), 9547–9561. 10.1021/acs.jctc.4c01021. 39428623 PMC11562374

[ref44] Perdew J. P.; Burke K.; Ernzerhof M. Generalized Gradient Approximation Made Simple. Phys. Rev. Lett. 1996, 77 (18), 3865–3868. 10.1103/PhysRevLett.77.3865. 10062328

[ref45] Tkatchenko A.; Scheffler M. Accurate Molecular Van Der Waals Interactions from Ground-State Electron Density and Free-Atom Reference Data. Phys. Rev. Lett. 2009, 102 (7), 073005 10.1103/PhysRevLett.102.073005. 19257665

[ref46] Luber S.; Iannuzzi M.; Hutter J. Raman Spectra from Ab Initio Molecular Dynamics and its Application to Liquid S-Methyloxirane. J. Chem. Phys. 2014, 141 (9), 094503 10.1063/1.4894425. 25194377

[ref47] Gonze X.; Vigneron J.-P. Density-Functional Approach to Nonlinear-Response Coefficients of Solids. Phys. Rev. B 1989, 39 (18), 13120 10.1103/PhysRevB.39.13120. 9948209

[ref48] Gonze X. Adiabatic Density-Functional Perturbation Theory. Phys. Rev. A 1995, 52 (2), 1096 10.1103/PhysRevA.52.1096. 9912349

[ref49] Kühne T. D.; et al. CP2K: An Electronic Structure and Molecular Dynamics Software Package-Quickstep: Efficient and Accurate Electronic Structure Calculations. J. Chem. Phys. 2020, 152 (19), 194103 10.1063/5.0007045. 33687235

[ref50] Goedecker S.; Teter M.; Hutter J. Separable Dual-Space Gaussian Pseudopotentials. Phys. Rev. B 1996, 54 (3), 1703 10.1103/PhysRevB.54.1703. 9986014

[ref51] Hartwigsen C.; Goedecker S.; Hutter J. Relativistic Separable Dual-Space Gaussian Pseudopotentials from H to Rn. Phys. Rev. B 1998, 58 (7), 3641 10.1103/PhysRevB.58.3641. 9986014

[ref52] Krack M. Pseudopotentials for H to Kr Optimized for Gradient-Corrected Exchange-Correlation Functionals. Theor. Chem. Acc. 2005, 114 (1), 145–152. 10.1007/s00214-005-0655-y.

[ref53] VandeVondele J.; Hutter J. Gaussian Basis Sets for Accurate Calculations on Molecular Systems in Gas and Condensed Phases. J. Chem. Phys. 2007, 127 (11), 114105 10.1063/1.2770708. 17887826

[ref54] Feuerstein L.; Bas E. E.; Heine T.; Golze D.; Oschatz M.; Weidinger I. Nitrile Groups as Build-in Molecular Sensors for Interfacial Effects at Electrocatalytically Active Carbon-Nitrogen Materials. 2024. 10.5281/zenodo.13354607. PMC12022941 40200634

