# Supplementary material for: Nitrile Groups as Build-In Molecular Sensors for Interfacial Effects at Electrocatalytically Active Carbon–Nitrogen Materials
Source: ACS Appl Mater Interfaces. 2025 Apr 9;17(16):23996–4004. doi: 10.1021/acsami.5c02366 (PMC12022941; doi:10.1021/acsami.5c02366)
Supplement: Supplementary file 1 — am5c02366_si_001.pdf [file am5c02366_si_001.pdf]

Supporting Information

Nitrile Groups as Build-in Molecular Sensors for Interfacial Effects at Electrocatalytically Active Carbon-Nitrogen Materials

*Linda Feuerstein[a], Ekin Esme Bas[b,c,d], Dorothea Golze[b], Thomas Heine[b,c,d,e], Martin Oschatz[f,g,h], Inez M. Weidinger[a]**

[a] Chair of Electrochemistry, Technische Universität Dresden, Zellescher Weg 19, 01069 Dresden, Germany
[b] Chair of Theoretical Chemistry, Technische Universität Dresden, Bergstrasse 66c, 01069 Dresden, Germany
[c] Helmholtz-Zentrum Dresden-Rossendorf, HZDR, Bautzner Landstrasse 400, 01328 Dresden, Germany
[d] Center for Advanced Systems Understanding, CASUS, Untermarkt 20, 02826 Görlitz, Germany
[e] Department of Chemistry, Yonsei University and ibs-cnm, Seodaemun-gu, Seoul 120-749, Republic of Korea
[f] Center for Energy and Environmental Chemistry, Friedrich Schiller University Jena, Philosophenweg 7a, 07743 Jena, Germany.
[g] Institute for Technical Chemistry and Environmental Chemistry, Friedrich Schiller University Jena, Philosophenweg 7a, 07743 Jena, Germany.
[h] Helmholtz Institute for Polymers in Energy Applications Jena (HIPOLE Jena), Lessingstraße 12–14, 07743 Jena, Germany.

**Corresponding author:* Inez M. Weidinger, E-mail: inez.weidinger@tu-dresden.de

Additional Raman and ATR-FTIR Spectra

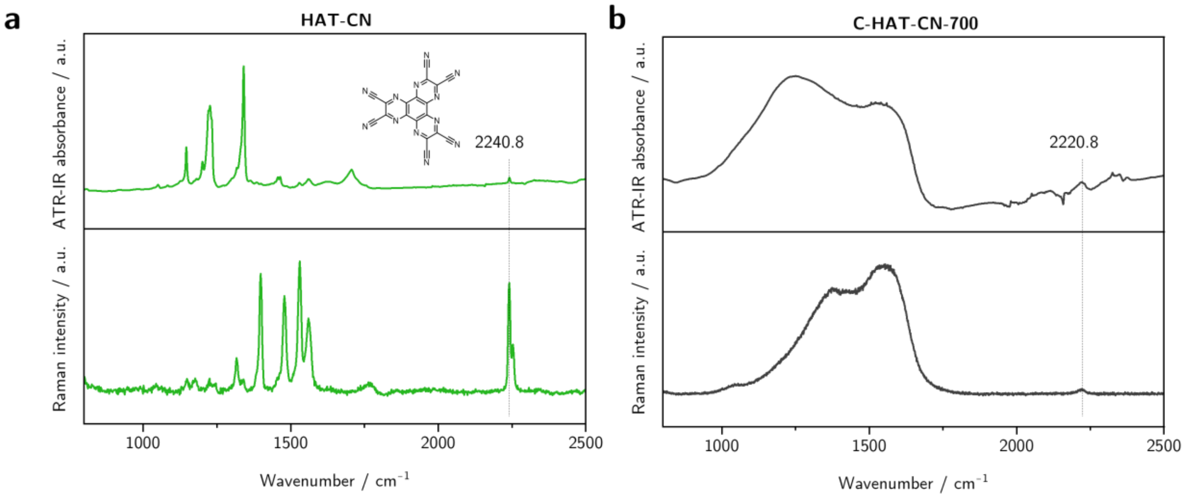

Figure S1: Experimental Attenuated Total Reflectance (ATR) IR spectra (top) and Raman spectra (bottom) of a HAT-CN, and b C-HAT-CN-700. Raman spectra of HAT-CN were measured at 640nm excitation wavelength, and C-HAT-CN-700 at 405nm excitation wavelength.

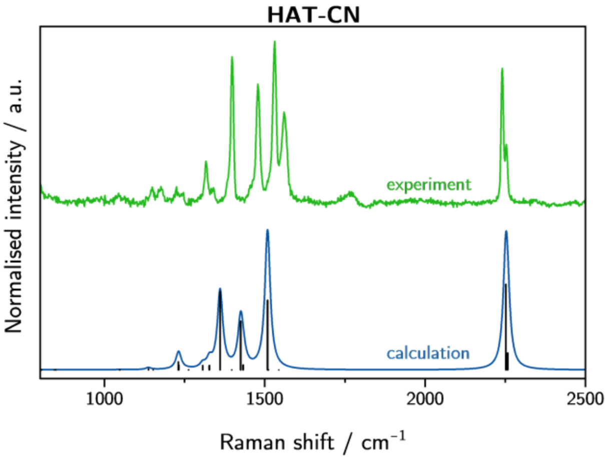

Figure S2: Experimental (top) and calculated (bottom) Raman spectra of the precursor material HAT-CN.

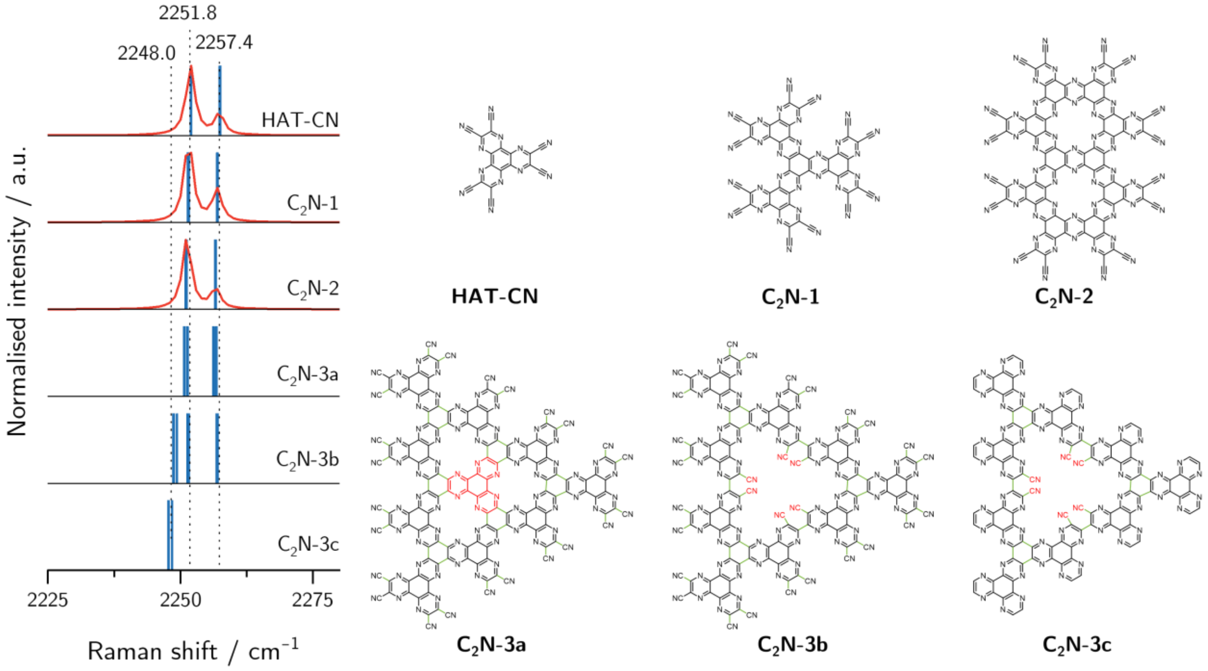

Figure S3: Calculated vibrational modes (blue bars) of model structures with varying degree of condensation (HAT-CN, C_2N-1, C_2N-2 and C_2N-3) and with "outer" and/or "inner" nitrile moieties (C_2N-3a-c). Raman spectra (red solid lines) are calculated for the structures HAT-CN, C_2N-1 and C_2N-2. We note that the position of the bands is determined in the normal mode analysis. Comparing the normal modes and the Raman spectra, only the intensities change; the position of the bands is the same.

Chronoamperometry in KCl

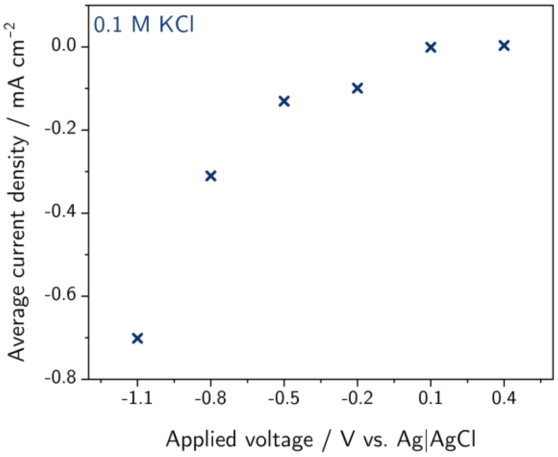

Figure S4: Chronoamperometric response currents measured during *in-situ* Raman measurements in 0.1 M KCl (average value of the last 10 % of the measurement time).

Dihedral Angles at Model Structure C_2N-3b

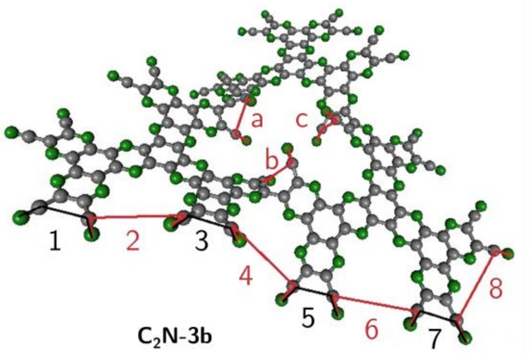

N-C-C-N dihedral angles			
"Outer" CN groups		"Inner" CN groups	
1	1.1°	a	97.0°
2	45.8°	b	95.4°
3	0.3°	c	97.0°
4	71.2°		
5	0.2°		
6	35.1°		
7	1.3°		
8	0.1°		

Figure S5: Overview of dihedral angles between neighbouring CN groups for the model structure C_2N-3b, containing "inner" and "outer" CN groups.

ν(C≡N) at Different KCl Concentrations

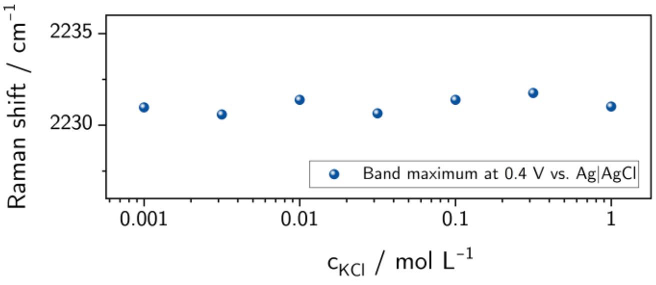

Figure S6: Position of ν(C≡N) peak in different KCl concentrations.

Linear Sweep Voltammetry (LSV) in HCl Electrolyte

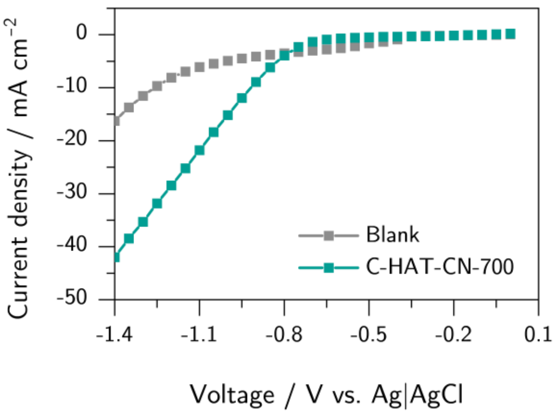

Figure S7: LSV curve of C-HAT-CN-700 and blank carbon paper in 0.1 M HCl at 50 mV s^{-1} scan rate.

ν(C≡N) During Chronoamperometry in HCl for 2h

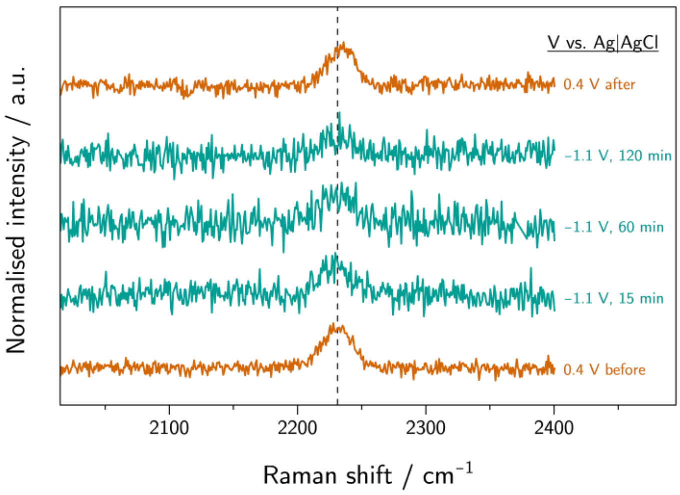

Figure S8: Raman spectra displaying the ν(C≡N) peak measured at 0.4 V, during chronoamperometry at −1.1 V over 120 min, and again afterwards at 0.4 V.

ν(C≡N) Peak Shift After Catalysis

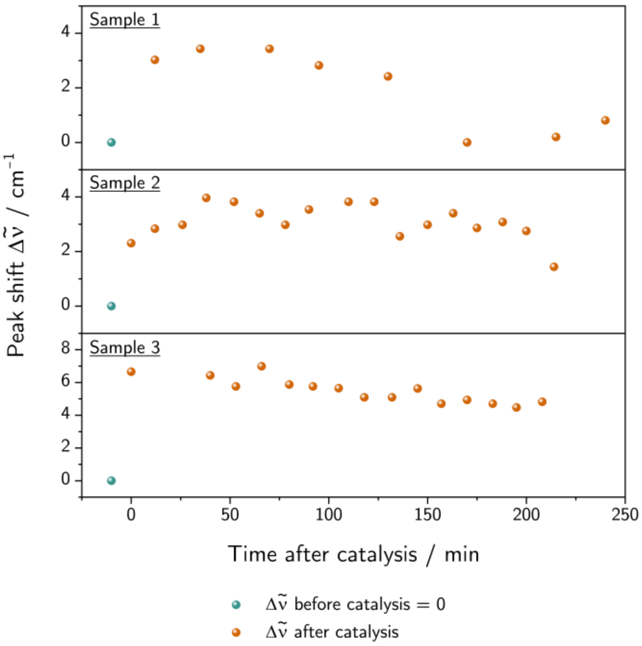

Figure S9: ν(C≡N) peak shift after HER catalysis measured at 0.4 V over time.

Raman Spectra of Dry C-HAT-CN-700 Electrode

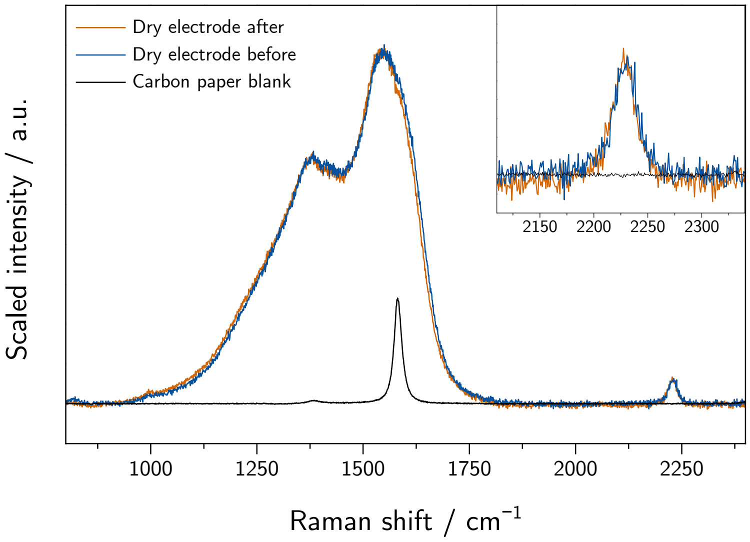

Figure S10: Raman spectra of dry C-HAT-CN-700 on carbon paper support electrode, before and after the electrochemical treatment. Note that deviations in the Raman spectra between 1250 – 1750 cm^{-1} might be due to underlying bands from the carbon paper that – depending on the Raman focus – contribute to the Raman spectra in different ratios.

ν(C≡N) at Different HCl Concentrations

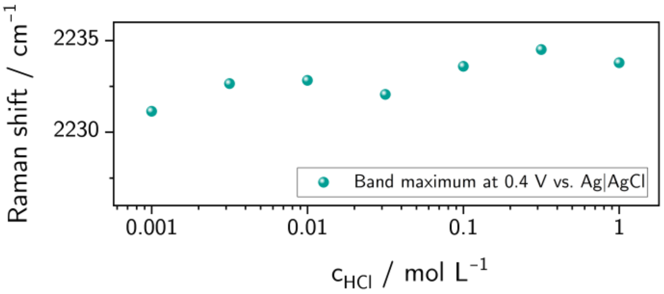

Figure S11: Position of ν(C≡N) peak in different HCl concentrations.
